# Isolation and structure determination of a tetrameric sulfonyl dilithio methandiide in solution based on crystal structure analysis and ^6^Li/^13^C NMR spectroscopic data

**DOI:** 10.3762/bjoc.16.172

**Published:** 2020-08-21

**Authors:** Jürgen Vollhardt, Hans Jörg Lindner, Hans-Joachim Gais

**Affiliations:** 1 DSM Nutritional Products, Wurmisweg 576, 4303 Kaiseraugst, Switzerland; 2 Clemens-Schöpf Institute of Organic Chemistry, Technische Universität Darmstadt, Alarich-Weiss-Straße 4, 64287 Darmstadt, Germany,; 3 Institute of Organic Chemistry, RWTH Aachen University, Landoltweg 1, 52074 Aachen, Germany

**Keywords:** crystal structure, dilithio sulfonyl methandiide, NMR, solution structure, X-ray analysis

## Abstract

Dilithio sulfonyl methandiides are a synthetically and structurally highly interesting group of functionalized geminal dianions. Although very desirable, knowledge of the structure of dilithio methandiides in solution was lacking up to now. Herein, we describe the isolation and determination of the structure of tetrameric dilithio (trimethylsilyl)(phenylsulfonyl) methandiide in solution and in the crystal. The elucidation of the structure of the tetramer is based on crystal structure analysis and ^13^C/^6^Li NMR spectroscopic data. A characteristic feature of the structure of the tetramer is the *C*_2_ symmetric C–Li chain, composed of four doubly Li-coordinated dianionic carbon and five Li atoms. Three Li atoms are devoid of a contact to a dianionic C atom. The tetramer, the dianionic C atoms of which undergo fast exchange, is in THF solution in fast equilibrium with a further aggregate, which is stable only at low temperatures.

## Introduction

**Figure 1:**
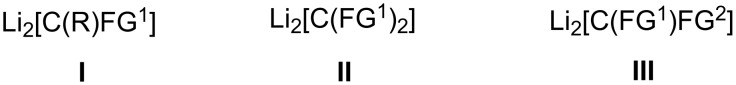
Functionalized dilithio methandiides (FG = functional group).

Functionalized dilithio methandiides **I–III** (Figure 1) are a fascinating class of compounds, whose reactivity, synthetic potential, and structure have received much interest in organic and metalorganic chemistry [1–2]. The most interesting aspects of compounds **I–III** are the likelihood of the formation of two new carbon–carbon and carbon–metal bonds, and the bonding situation of the dianionic carbon atom, including its electronic structure and coordination geometry, together with the possibility of a coordination by two lithium atoms.

The sulfonyl dilithio methandiides **2**, carrying various substituents R^1^ and R^2^, have attracted particular attention [1–6]. Reactivity studies of **2** with carbon and metal-based electrophiles revealed a high synthetic potential, allowing, for example, the synthesis of carbo- and heterocycles [4,6], and transition-metal carbene complexes [1–2], carrying the synthetically versatile sulfonyl group [7–8].

**Scheme 1:**
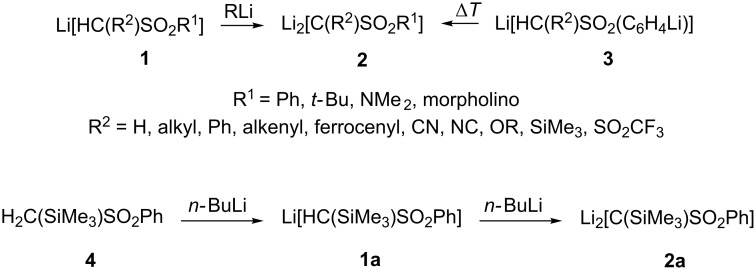
Synthesis of sulfonyl dilithio methandiides **2**.

Dilithio methandiides **2** are accessible from sulfonyl lithio methanides **1** [9] through α-deprotonation [1–2,4], and from arylsulfonyl dilithio methanides **3** [10–11] though *ortho*,α-transmetallation [10–13] (Scheme 1).

We had found that the α-deprotonation of the lithio methanide **1a** with *n*-butyllithium (*n*-BuLi) gave the stable silyl and sulfonyl-substituted dilithio methandiide **2a** [14–15]. The use of *n-*BuLi, inadvertently containing Li_2_O, had yielded prismatic crystals of **2a**. An X-ray crystal structure analysis had shown a *C*_i_ symmetric hexamer, (**2a**)_6_·Li_2_O·(THF)_6_, the dianionic C atoms of which are each coordinated by two Li atoms in a non-planar fashion (vide infra) [15]. Because of the general interest in dilithio methandiides that remains unabated to this day, crystal structure analysis has been carried out of a large number of derivatives [16–43]. They generally showed intricate aggregates with complex C–Li and C–Li–heteroatom chains, containing doubly lithiated C atoms. However, as much as there is now knowledge of the structure of dilithio methandiides in the crystal, as little is known about the structure and dynamics in solution [1–2,16–43]. The main obstacles at characterizing dilithio methandiides in terms of aggregate size, C–Li connectivity, Li coordination of the dianionic C atoms, and dynamics were poor solubility and problems to locate the ^13^C signals of the dianionic C atoms or the detection of only broad ones. Knowledge of the solution structure of dilithio methandiides would be, however, highly desirable in order to obtain a more complete understanding of the reactivity and coordination chemistry in general, and of the dianionic C atom in particular. During the structural investigation of **2a** it was observed that the Li_2_O-free methandiide is in contrast to (**2a**)_6_·Li_2_O·(THF)_6_ readily soluble in THF. ^6^Li and ^13^C NMR spectroscopy of **2a** had led to the detection of an aggregate, a substructure of which could be disclosed [15].

We describe in this paper the isolation of a tetramer of **2a** and the determination of its structure in solution based on crystal structure analysis and previously described NMR spectroscopic data.

## Results and Discussion

### Crystal structure

**Figure 2:**
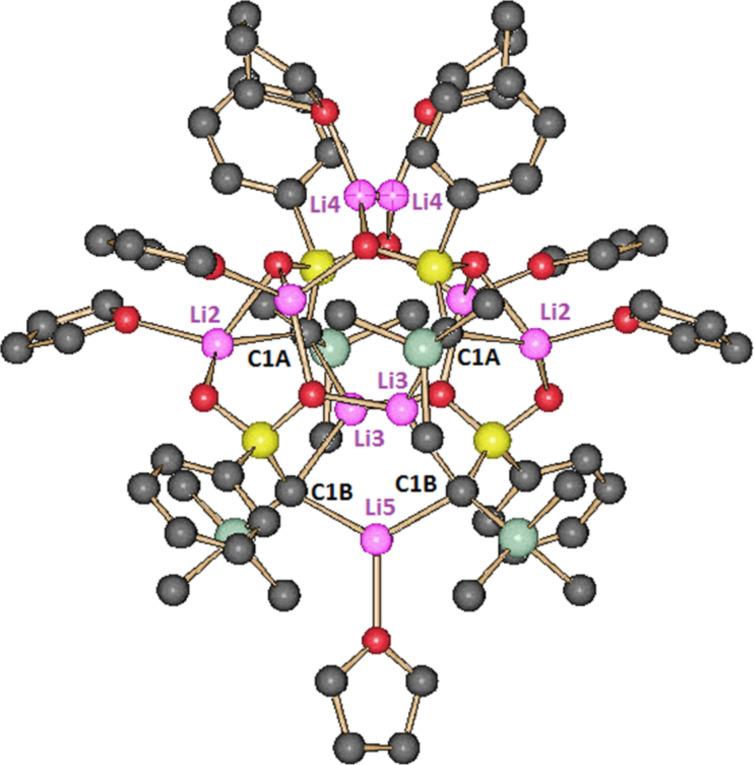
Structure of (**2a**)_4_·(THF)_6_ in the crystal. Color code: black, C; green, Si; yellow, S; red, O; pink, Li. Hydrogen atoms are omitted for clarity. Lithium atoms Li1 are unmarked because of a lack of space.

Treatment of sulfone **4** in a mixture of THF and *n*-hexane with Li_2_O-free *n*-BuLi (1.88 equiv) in *n*-hexane at −90 °C followed by warming the solution to room temperature gave octahedral crystals of **2a**. The use of two equivalents of the base led two a slower crystal growth. The X-ray crystal structure analysis showed a chiral *C*_2_ symmetric tetramer, (**2a**)_4_·(THF)_6_, containing six THF molecules (Figure 2) [44]. The lithium atom Li4 is not exactly located on the *C*_2_ axis. It is resolved by two Li4 in general positions near the *C*_2_ axis each with an occupancy of 0.5. Therefore, Figure 2 shows two positions for Li4 and the attached THF molecule (see the Supporting Information File 1 for details). The aggregate has two different types of dianionic carbon atoms, C1A and C1B, which are each coordinated by two Li atoms. Five Li atoms are coordinated by carbon atoms (2 Li2, 2 Li3 and Li5) and three Li atoms are without contact to a carbon atom and only coordinated by oxygen atoms (2 Li1 and Li4). Lithium atoms Li3 and Li5, which are each coordinated by two dianionic carbon atoms, have planar trigonal coordination geometry. The coordination geometry of C1A and C1B is characterized by τ_4_ values [45] of 0.68 and 0.86, respectively, indicating seesaw geometry for C1A and a trigonal pyramidal one for C1B.

**Figure 3:**
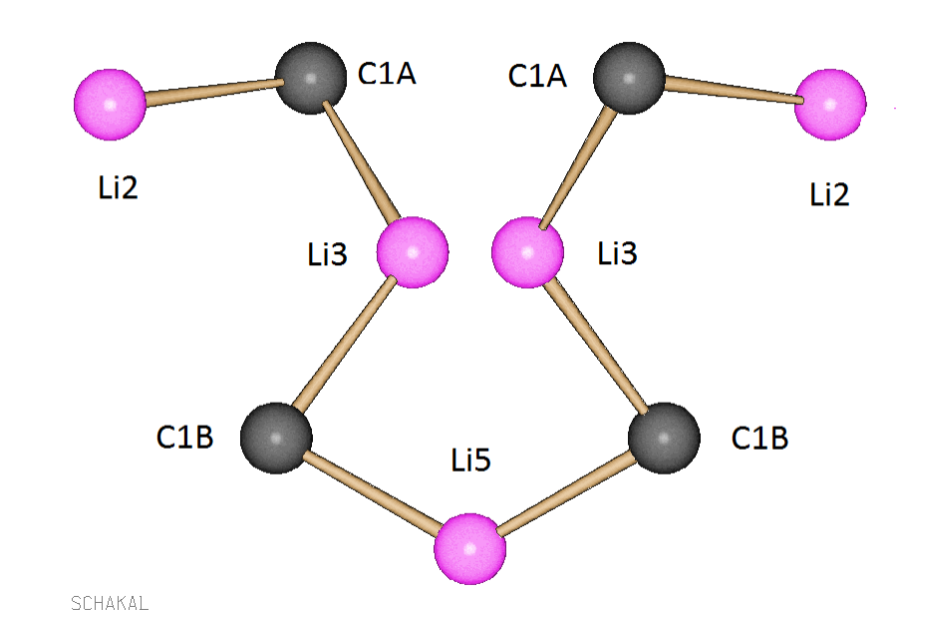
The *C*_2_ symmetric carbon–lithium chain of aggregate (**2a**)_4_·(THF)_6_ in the crystal (color code: black, C; pink, Li). All atoms except C1A, C1B, Li2, Li3, and Li5 are omitted for clarity.

The most interesting structural feature of tetramer (**2a**)_4_·(THF)_6_ is the linear carbon–lithium chain shown in Figure 3, which has *C*_2_ symmetry and contains the four dianionic carbon atoms and five lithium atoms. It will be shown later that the presence of this chain is the key to the assignment of the structure of the aggregate in solution (vide infra).

The tetramer (**2a**)_4_·(THF)_6_ shows besides the C–Li chain a number of further interesting structural features. Lithium atom Li5 bridges an eight-membered (C–Li–O–S)_2_ ring at the carbon atoms, while Li4 overpasses an eight-membered (O–S–O–Li)_2_ ring at the oxygen atoms. Eight-membered (O–S–O–Li)_2_ rings of this type are also found as typical structural element of dimeric sulfonyl lithio methanides **1** in the crystal [46–50]. The atoms C1A, Li2, O1A, and S1 are embedded in two four-membered rings, while two lithium atoms, a sulfur atom, and three oxygen atoms form two six-membered rings. Because of the presence of C–Li and O–Li bonds in (**2a**)_4_·(THF)_6_, the dicarbanions are both C,C- and O,Li-dilithiated species.

The C–Li, C–S, and O–Li bond lengths (Table 1) of the tetramer (**2a**)_4_·(THF)_6_ are in the range of those found in the hexamer (**2a**)_6_·Li_2_O·(THF)_6_ and functionalized dilithio sulfonyl methandiides [40,43].

**Table 1:** Selected bond lengths, bond angles, and dihedral angles of (**2a**)_4_·(THF)_6._

bond lengths^a^	bond angles^b^

C1A–S 156.8(6)	C1A-S-C2A 115.4(3)
C1B–S 158.7(6)	C1A–Li3–C1B 120.9(5)
C1A–Li2 222.2(11)	O1F–Li5–C1B 118.6(4)
C1A–Li3 222.7(11)	C1B–Li5–C1B 122.8(7)
C1B–Li3 224.2(11)	O2B–Li3–C1A 117.2(5)
C1B–Li5 209.8(9)	O2B–Li3–C1B 121.4(5)
O1–Li2 216.7(12)	S-C1A-Si 125.4(4)
O1–Li1 196.7(10)	C1B-S-C2B 115.3(3)
O2–Li4 187(4)	Li2–C1A–Li3 86.2(4)
O1B–Li2 189.1(11)	S-C1A-Li2 85.2(4)
O2B–Li1 195.6(10)	Li5–C1B–Li3 91.4(4)
C1A–Si 179.3(6)	S-C1B-Si 117.9(3)
C1B–Si 180.9(6)	

dihedral angles^b^	

Li2–C1A–Li3–C1B 35.2(6)	C2A-S-C1A-Si 51.2(5)
Li3–C1B–Li5–C1B 33.6(3)	C2B-S-C1B-Si 78.6(4)

^a^In pm. ^b^In degree.

### Solution structure

The synthesis of **2a** from sulfone ^6^Li and ^13^C-labelled **4** with Li_2_O-free *n*-BuLi had yielded the methandiide, being readily soluble in THF. ^13^C and ^6^Li NMR spectroscopy of ^13^C,^6^Li-**2a** in [D_8_]THF [15,51–53] had shown an aggregate, **2a**-**I**, which is present in the whole temperature range from 22 °C to −103 °C. The formation of a further equilibrium aggregate, **2a**-**II**, was detected from −50 °C downwards. The equilibrium between the aggregates lies at −103 °C on the side of **2a**-**II**. Interestingly, formation of **2a**-**II** in THF could not be detected when diglyme was present.

**Figure 4:**
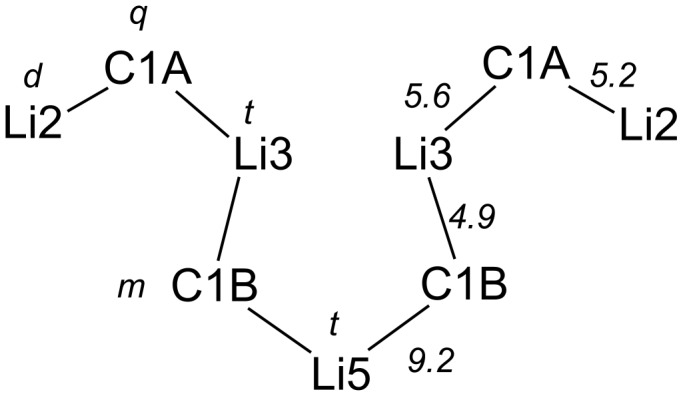
*C*_2_ Symmetric carbon–lithium chain of **2a**-**I** in THF, showing the carbon–lithium connectivities, multiplicities of the NMR signals (left), and magnitudes of ^1^*J*(^6^Li,^13^C) couplings in Hz (right) [15].

According to the ^13^C and ^6^Li NMR spectroscopic experiments of **2a**-**I** at −103 °C, the aggregate contains a *C*_2_ symmetric linear carbon–lithium chain composed of the four dianionic carbon and five lithium atoms (Figure 4).

The carbon–lithium chain includes three different types of lithium atoms, Li2, Li3, and Li5 and two different types of dianionic atoms, C1A and C1B. The ^13^C NMR spectrum of ^13^C,^6^Li-**2a**-**I** at −103 °C showed a quintet (*q*) for C1A (δ = 49.9 ppm) and a multiplet (*m*) for C1B (δ = 51.4 ppm) due to ^6^Li,^13^C spin coupling. The ^6^Li NMR spectrum of **2a**-**I** at −103 °C displayed three signals split by ^6^Li,^13^C spin coupling. Selective ^6^Li{^13^C} double resonance and two-dimensional double quantum based shift correlation experiments had served to establish the ^6^Li–^13^C connectivity. In addition, ^6^Li spin echo spectroscopy with gated ^13^C decoupling had confirmed the multiplicities of the ^6^Li signals. All together these experiments had established a triplet (*t*) structure for the signals of Li3 (δ = 2.62 pm) and Li5 (δ = 2.86 pm) and a doublet (*d*) structure for the signal of Li2 (δ = 1.17 pm) [15,51–53]. Essential for the success of the NMR spectroscopic investigation of **2a** was the ^13^C,^6^Li labelling of the dilithio methandiide. It allowed the detection of the otherwise low intensity signals of the dianionic carbon atoms of the aggregates and the attainment of signals with a small line width [54].

The complete connectivity of the carbon–lithium chain of **2a**-**II** could not be revealed, since the determination of the multiplicities of all ^6^Li and ^13^C signals failed.

Undoubtedly, the *C*_2_ symmetric linear C–Li chain is the most revealing structural feature of tetramer **2a**-**I**, since it contains besides Li atoms the four dianionic C atoms. A chain having these carbon–lithium connectivities is contained in tetramer (**2a**)_4_·(THF)_6_ in the crystal. The excellent agreement between the two C–Li chains shows that **2a**-**I** is a tetramer, adopting in solution the same structure as the tetramer (**2a**)_4_·(THF)_6_ in the crystal. Although the lithium oxygen bonds of **2a**-**I** evaded a direct detection by NMR spectroscopy, the ^13^C,^6^Li shift correlation experiments of **2a**-**I** had shown two ^6^Li signals, lacking correlation with ^13^C signals [15]. Thus, there is good reason to believe that the lithium oxygen bonds of (**2a**)_4_·(THF)_6_ are retained in **2a**-**I** in solution. Most likely, six lithium atoms of **2a**-**I** are coordinated by THF molecules in a similar way as in tetramer (**2a**)_4_·(THF)_6_.

The aggregate **2a**-**I** is fluxional in THF with an estimated barrier of Δ*G*^≠^ = 12.0 ± 0.5 kcal/mol (248 K) for the exchange of the dianionic C atoms C1A and C1B [53]. An intra-aggregate exchange seems to be less probable, because it would require an extensive carbon lithium and oxygen lithium bond reorganization (cf. Figure 2). A more likely scenario for the exchange would be the existence of a THF-assisted tetramer–dimer equilibrium.

### Bonding of the dianionic C atoms

^6^Li NMR spectroscopy of tetramer **2a**-**I** had revealed a dianionic carbon atom, C1B, carrying two different Li atoms, a bonding situation which is reflected by the two different ^6^Li,^13^C coupling constants. It was thus of particular interest to see whether this feature is mirrored by the bonding situation of C1B of (**2a**)_4_·(THF)_6_. An inspection of the tetramer shows for C1B different carbon–lithium bond lengths, while those of C1A are similar in length. The shorter C1B–Li5 bond corresponds to a larger and the longer C1B–Li3 bond to a smaller ^6^Li,^13^C coupling constant (Table 2). Accordingly, the similar coupling constants of C1A in **2a**-**I** are matched by similar carbon lithium bond lengths of C1A in (**2a**)_4_·(THF)_6_. It is generally accepted that the carbon lithium interaction in dilithio methandiides is ionic in nature [1–2]. The observation of ^6^Li,^13^C couplings shows, however, that the carbon lithium bonds in **2a**-**I** have a covalent contribution as generally observed for organolithiums [55].

**Table 2:** ^1^*J*(^13^C,^6^Li) coupling constants of **2a**-**I** and bond lengths of (**2a**)_4_·(THF)_6_.

bond	**2a**-**I**	(**2a**)_4_·(THF)_6_
	^1^*J*^a^	lengths^b^

C1B–Li5	9.2	209.8(11)
C1B–Li3	4.9	224.2(11)
C1A–Li3	5.2	222.7(11)
C1A–Li2	5.6	222.2(11)

^a^In Hz. ^b^In pm.

Three mechanisms most likely contribute to the stabilization of the negative charge of the carbon atoms of tetramer (**2a**)_4_·(THF)_6_ and hexamer (**2a**)_6_·Li_2_O·(THF)_6_, electrostatic interaction with and charge polarization by the positively charged sulfur atom [56] and silicon atom [57], and negative *n*_C_–σ*_SPh_ hyperconjugation [56,58]. The anions of (**2a**)_4_·(THF)_6_ and (**2a**)_6_·Li_2_O·(THF)_6_ adopt conformations around the carbon sulfur bond, which would allow for stabilization by negative hyperconjugation. Density functional theory and natural bond order calculations of dilithio methandiides of type **II** [32,43] suggest that the two lone pairs at the dianionic carbon atom of (**2a**)_4_·(THF)_6_ and (**2a**)_6_·Li_2_O·(THF)_6_ are perhaps located in an sp^2^ hybridized orbital and p orbital. However, the importance and quantification of the three stabilizing factors as well as the electronic structure of **2a** have to await investigation by quantum chemical calculation. It seems appropriate in this context to have a look at the bonding situation of the corresponding sulfonyl lithio methanides **1**. Their anion is stabilized by electrostatic interaction, polarization, and negative *n*_C_-σ*_SR_ hyperconjugation, with electrostatic interaction as dominating mechanism [56,58]. The lithio methanides **1** are monomeric or dimeric in THF solution and the solid state, depending on Lewis basic ligands of the lithium atom [46–50]. A characteristic feature of **1** is the formation of O–Li bonds in solution and in the crystal. The NMR spectroscopic investigation of **1**, including **1a** [53], gave no indication for the existence of a species with a C–Li bond. However, the failure to detect a ^6^Li,^13^C coupling is no proof for the not existence of a C–Li-bonded species, because of the possibility of a fast C,Li exchange process, even at low temperatures. While the anion of **1** carries one lone pair at the carbon atom, the anion **2** bears two lone pairs. The higher negative charge density of the carbon atom of **2** seems to be a decisive factor for the formation of C–Li bonds in the crystal and in solution.

### Comparison of tetramer and hexamer

**Figure 5:**
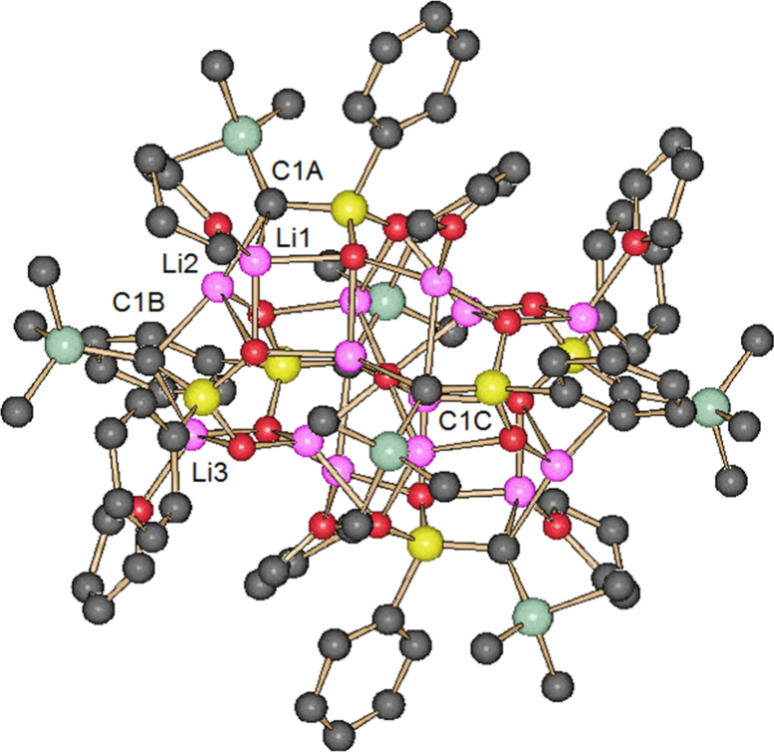
the structure of (**2a**)_6_·Li_2_O·(THF)_6_ in the crystal [15]. Color code: black, C; green, Si; yellow, S; red, O; pink, Li. Hydrogen atoms are omitted for clarity. Selected bond distances (pm) and dihedral angles (°): C1A–Li1 218, C1A–Li2 225, C1B–Li2 219, C1B–Li3 234, C1C–Li7 223, C1C–Li4 251, C1C–Li6 283, C2A–S–C1A–Si 50, C2B–S–C1B–Si 12, C2C–S–C1C–Si 58.

It seems interesting to compare the carbon lithium connectivity and lithium coordination of the dianionic carbon atoms of tetramer (**2a**)_4_·(THF)_6_ and Li_2_O-containing hexamer (**2a**)_6_^.^Li_2_O·(THF)_6_. The hexamer has an octahedral cluster of lithium atoms with an oxygen atom in the center of symmetry and three different types of strongly distorted tetrahedral dianionic carbon atoms (C1A: τ_4_ = 0.66, C1B: τ_4_ = 0.75, C1C: τ_4_ = 0.61) (Figure 5). It contains two linear Li1–C1A–Li2–C1B–Li3 chains and two dianionic carbon atoms, C1C, outside of the chains, which are each coordinated by three Li atoms. In hexamer (**2a**)_6_·Li_2_O·(THF)_6_ all lithium atoms are coordinated by dianionic carbon atoms, while three lithium atoms of tetramer (**2a**)_4_·(THF)_6_ are without contact to the dianionic C atoms. Interestingly, the structure of (**2a**)_6_·Li_2_O·(THF)_6_ is in contrast to that of (**2a**)_4_·(THF)_6_ characterized by a large number of four-membered Li–C–S–O and Li–O–Li–O chelate rings. The dianions of hexamer (**2a**)_6_·Li_2_O·(THF)_6_ are as those of the tetramer both C,C- and O,Li-dilithiated species.

### Comparison of (**2a**)_4_·(THF)_6_ and (**2a**)_6_·Li_2_O·(THF)_6_ with functionalized sulfonyl dilithio methandiides

Besides the crystal structures of (**2a**)_4_·(THF)_6_ and (**2a**)_6_·Li_2_O·(THF)_6_ those of the sulfonyl dilithio methandiides **IIIa** (FG^1^ = SO_2_Ph, FG^2^ = P(S)Ph_2_) [36] and **IIIb** ((FG^1^ = SO_2_Ph, FG^2^ = P(NSiMe_3_)Ph_2_) [43] have been determined. Methandiides **IIIa** and carbanion **IIIb** have in contrast to **2a** a further lithium atom coordinating and stabilizing heteroatom-substituent. Both methandiides are tetramers, featuring dilithiated dianionic carbon atoms with a coordination geometry, which strongly deviates from tetrahedral and O–Li, S–Li, and N–Li bonds. They are, however, devoid of C–Li chains of the type found in (**2a**)_4_·(THF)_6_ and (**2a**)_6_·Li_2_O·(THF)_6_. The bonding situation of tetramer (**2a**)_4_·(THF)_6_ and hexamer (**2a**)_6_·Li_2_O·(THF)_6_ indicates that the dominating structure building factor is the interaction between the charged carbon, oxygen and lithium atoms and not between orbitals. A similar conclusion was reached in the case of the dilithio methandiides **IIIa** and **IIIb**.

## Conclusion

The determination of the structure of tetramer **2a**-**I** in both the solution and solid state, which is the first of a dilithio methandiide, was achieved based on X-ray crystal structure analysis and ^6^Li,^13^C NMR spectroscopic data. The tetramer **2a**-**I** in THF solution has the same structure as the tetramer (**2a**)_4_·(THF)_6_ in the crystal. A characteristic feature of the structure of the tetramer is the *C*_2_ symmetric linear C–Li chain, composed of the four dianionic C atoms and five Li atoms. Each dianionic C atom is coordinated by two Li atoms. While one dianionic C atom has seesaw coordination geometry, the other dianionic C atom has a trigonal bipyramidal one. As a consequence of the C–Li chain three Li atoms are without a contact to a dianionic C atom. The tetramer is fluxional leading to an exchange of the dianionic C atoms. It stands in THF in equilibrium with a second aggregate, which is only stable at low temperatures.

## Supporting Information

File 1 Experimental details, crystal data, and parameters of data collection for tetramer (**2a**)_4_^.^(THF)_6_.
