# Supplementary material for: Isolation and structure determination of a tetrameric sulfonyl dilithio methandiide in solution based on crystal structure analysis and 6Li/13C NMR spectroscopic data
Source: Beilstein J Org Chem. 2020 Aug 21;16:2057–63. doi: 10.3762/bjoc.16.172 (PMC7445413; doi:10.3762/bjoc.16.172)
Supplement: File 1 — Experimental details, crystal data, and parameters of data collection for tetramer (2a)4.(THF)6. [file Beilstein_J_Org_Chem-16-2057-s001.pdf]

BEILSTEIN JOURNAL OF ORGANIC CHEMISTRY

Supporting Information

for

Isolation and structure determination of a tetrameric sulfonyl dilithio methandiide in solution based on crystal structure analysis and $^{6}Li/^{13}C$ NMR spectroscopic data

Jürgen Vollhardt, Hans Jörg Lindner and Hans-Joachim Gais

Beilstein J. Org. Chem. **2020,** *16,* 2057–2063. doi:10.3762/bjoc.16.172

Experimental details, crystal data, and parameters of data collection for tetramer $(2a)_4 \cdot (THF)_6$

License and Terms: This is a supporting information file under the terms of the Creative Commons Attribution License (https://creativecommons.org/licenses/by/4.0). Please note that the reuse, redistribution and reproduction in particular requires that the authors and source are credited.
The license is subject to the *Beilstein Journal of Organic Chemistry* terms and conditions: (https://www.beilstein-journals.org/bjoc)

Table of contents

1. Isolation of single crystals of (**2a**)$_4$·(THF)$_6$ S2
2. Crystal data and parameters of data collection for (**2a**)$_4$·(THF)$_6$..................... S3

1. Isolation of single crystals of (2a)$_4$·(THF)$_6$

General: All experiments were carried out by using Schlenk and syringe techniques in flame-dried glassware under argon. THF and *n*-hexane were purified by distillation from potassium benzophenone ketyl. Li_2O-free *n*-BuLi was prepared as follows. To *n*-BuLi (50.1 mL of 1.67 M in *n*-hexane) was added at room temperature MeI (0.30 mL, 4.85 mmol). The mixture became turbid after a few seconds. Then, a colorless solid deposited and the initial yellow color disappeared. After the mixture was kept for 24 h at room temperature, the clear and nearly colorless supernatant was removed by a syringe. Titration of an aliquot with $(Ph)_2CHCO_2H$ showed a 1.33 M solution of *n*-BuLi.

Experimental: To a solution of sulfone **4** (225 mg, 0.987 mmol) in a mixture of THF (2.50 mL) and *n*-hexane (0.60 mL) was added at −90 °C Li_2O-free *n*-BuLi (1.40 mL of 1.33 M in *n*-hexane). The flask was closed, the cooling bath removed, and the mixture warmed without vibration to 22 °C (air bath). After 20 min, thirty to forty clear, strongly refractive octahedral crystals of (**2a**)$_4$·(THF)$_6$ had formed. Most of the crystals developed cracks from the outside to the inner side. Crystallization was interrupted by replacement of the mother liquor by a mixture of *n*-hexane and THF (10 mL, 30:1). Then, an intact crystal was transferred to a Mark tube, which was sealed with a burner.

2. Crystal data and parameters of data collection for $(2a)_4{\cdot}(THF)_6$

A crystal of 0.22 × 0.30 × 0.35 mm was found to be suitable for single crystal X-ray structure determination. A total number of 5736 reflections (including 3075 with $I > 2\sigma(I)$) were collected in the $\omega/2\Theta$ mode at 293 K employing MoKα radiation (λ = 0.71073 Å) within the limits of Θ_{min} = 4.91 and Θ_{max} = 23.08°. Cell dimensions of a = 21.287(9) Å, b = 18.734(8) Å, and c = 20.928(9) Å result in a cell volume of 8346(6) $Å^3$ and a calculated density of 1.166 g cm^{-3}. The absorption coefficient was found to be 0.226 mm^{-1}, no absorption correction. T_{min} = 0.924, T_{max} = 0.951. The tetramer crystalizes in orthorhombic space group *Pbcn*(60). The structure was both solved by direct methods and refined employing the SHELXS-97 suite of crystallographic routines. Refinement of 485 parameters against F^2 including all reflections resulted in a R-factor of w_r = 0.2537 and an error of fit of 1.032. The residual electron density at this point was 0.571/−0.385. Refinement against F resulted in R-factors of 0.1653 (including all reflections, where negative F were set to zero) and of 0.069 including only those with $F^2 > 2\sigma(F^2)$. Part of the hydrogen positions could be located in Fourier difference maps where the remaining ones were calculated in idealized positions.

The tetramer (**2a**)$_4$·(THF)$_6$ sits on a crystallographic C_2 axis with a number of disorders. The methyl groups of a trimethylsilyl group are represented by two sets of C atoms each with occupancy of 0.5. Li4 is not exactly located on the C_2 axis. It is resolved by two Li4 in general positions near the C_2 axis each with an occupancy of 0.5. The THF molecule E is statistically distributed between two positions each with an occupancy of 0.5. The O atom was refined and the positions of the C atoms were obtained through Fourier synthesis. All H atoms except those in disordered areas were positioned following geometrical arguments. All atoms except H and Li4 and the C atoms of the THF molecule E were anisotropically refined.
